# Hardening Steel by Gas

**Published:** 1896-04

**Authors:** 


### Hardening Steel by Gas.

The Germans are interested in a new process for hardening steel by means of gas, says *The Engineering and Mining Journal* (March 7). The invention originated with the famous French steel and iron firm, Schneider & Co., of Creuzot. It is a well-known fact that gas, under great heat, deposits carbon in solid form. Upon this depends its light effects, and also the formation of the so-called retort graphites, a thick covering of pure carbon on the walls of the gaslight retorts. The gas that strikes the retort walls deposits part of its carbon upon them. This is the fact upon which Schneider bases his very useful invention—a process for cementing together (uniting) steel-armor plates. It is said to be very important in the production of armor plates to have them comparatively soft inside and hard outside. This hardening is obtainable by the application of carbon. Formerly, the process of hardening consisted in covering the plates with layers of coal and heating them till they glowed. Schneider's process puts two plates into a furnace, one on top of the other, with a hollow space between. This space is made gas-tight by means of asbestos packing put on around the edges, and the plates are heated red-hot, while a stream of light gas is poured into the hollow space indicated. The carbon thrown out by the gas is greedily taken up by the glowing plates until they are thickly covered. The depth of this carbon covering can be regulated by the amount of gas admitted. In order to secure regular and uniform action during the process, and to prevent the pipes that carry the gas to the hollow space from absorbing any of the carbon, they are insulated in other pipes through which water is constantly circulating. It is believed that this simple and rapid carbonizing process will soon be applicable to many other branches of the steel industry.

